# Associations Between Colorectal Cancer Screening and Glycemic Control in People With Diabetes, Boston, Massachusetts, 2005-2010

**Published:** 2011-06-15

**Authors:** Joanne E. Wilkinson, Larry Culpepper

**Affiliations:** Department of Family Medicine, Dowling 5, Boston University School of Medicine; Department of Family Medicine, Boston University School of Medicine, Boston, Massachusetts

## Abstract

**Introduction:**

Recent studies indicate an increased risk of colorectal cancer in people with diabetes. However, people with diabetes may have lower colorectal cancer screening rates than people without diabetes. Few data are available regarding factors associated with lack of screening for people with diabetes. Our objective was to describe factors associated with lack of timely colorectal cancer screening in people with diabetes.

**Methods:**

We examined an electronic medical record database with more than 6,000 patients aged 50 years or older who had diabetes and were seen in a large hospital system in Boston, Massachusetts. We compared patients who had received timely colorectal cancer screening with those who had not on several variables, including glycemic control, expressed as average hemoglobin A1c (HbA1c). Bivariate analyses were performed using χ^2^ and *t* tests for means when applicable. Logistic regression was used to determine the independent association of variables with lack of screening.

**Results:**

Patients with poor glycemic control (average HbA1c >8.5%) were more likely not to have been screened for colorectal cancer than those with good glycemic control, even after adjusting for the number of primary care visits. Patients with fewer than 20 primary care visits in 5 years were more likely not to have been screened than those with more visits.

**Conclusion:**

Glycemic control appears to be independently associated with the likelihood of colorectal cancer screening. People with poorly controlled diabetes should be targeted in future research and individual patient care.

## Introduction

Researchers have noted an increased risk of colorectal cancer and its precursors in people with diabetes (1-5). This increased risk is hypothesized to be secondary to several factors, such as increased circulating insulin and insulin-like growth factor (IGF-1), and to effects of treatment for diabetes (6-8). Awareness of this increased risk may encourage health care providers to promote colorectal cancer screening to patients with diabetes. Primary care physicians are the most likely candidates to recommend screening, since they provide care to the majority of diabetes patients nationally (9-11) and are primarily responsible for screening and prevention.

Rates of colorectal cancer screening in many states were below 50% between 1998 and 2005 (12), although more recent data indicate rates of 50% to 60% in most states and 60% to 70% in others (13). The US Preventive Services Task Force recommends colonoscopy every 10 years, sigmoidoscopy every 5 years, or annual fecal occult blood testing (FOBT) starting at age 50 and continuing until at least age 75 (14). Efforts are under way to increase rates of colorectal cancer screening among racial/ethnic minorities but have not targeted people with diabetes, even though their risk for colorectal cancer may be higher than the risk for the general population (15). People with diabetes may be less likely to pursue any cancer screening (16,17), even with more primary care visits per year than patients without diabetes (18).

Glycemic control may be associated with colorectal cancer screening in people with diabetes for several reasons. Many diabetic patients with good glycemic control (hemoglobin A1c [HbA1c] <7.0%) visit their physician regularly and adhere to lifestyle recommendations regarding diabetes; therefore, they would have more frequent opportunities to hear about screening and may be more likely to accept colorectal cancer screening recommendations. People with fair (HbA1c 7.0%-8.5%) or poor (HbA1c >8.5%) glycemic control may visit their physician less regularly and be less likely to adhere to prescribed dietary and medication regimens, and may be less likely to accept colorectal cancer screening recommendations.

However, even when receiving regular care, patients with poorly controlled diabetes may not be screened for colorectal cancer. They may have multiple medical and social issues for the physician to address at each visit, and a substantial amount of the visit may be devoted to improving glycemic control, leaving the physician unable to accomplish all preventive goals (19). These patients may also be more medically complex, with more diabetic complications, leading the physician to defer colorectal cancer screening until they are more stable.

The existing research on low rates of screening for other cancers in people with diabetes and on competing priorities in the office visit prompted us to examine associations between glycemic control and rates of colorectal cancer screening in people with diabetes. The objective of this study was to examine the association between glycemic control (average HbA1c) and colorectal cancer screening rates (colonoscopy or sigmoidoscopy within 5 years or FOBT within 1 year) in a large urban hospital with robust numbers of racial/ethnic minorities and large numbers of patients with poorly controlled diabetes. The findings from this study should add to physician awareness about colorectal cancer screening for people with diabetes by identifying their unique factors associated with lack of screening and generate hypotheses for future research.

## Methods

### Database

We conducted a secondary data analysis by using a large database that contained elements of de-identified electronic medical records at a large safety-net hospital in Boston, Massachusetts (historically, >80% of patients have had some form of government insurance, and >50% of the patient population comprises racial/ethnic minorities). Patients were included in the analysis if they were listed as having a diagnosis of diabetes, were aged 50 years or older on January 1, 2005, had a primary care physician at the hospital, and had at least 1 HbA1c value listed in the database between January 1, 2005, and January 1, 2010 (n = 6,066). Any patients who met these criteria were included, regardless of the number of HbA1c values charted or the number of primary care visits. Patients were excluded if they had a diagnosis of colorectal cancer. This project was designated as "exempt" by the institutional review board at Boston University School of Medicine/Boston Medical Center.

Most patients at this institution receive both their primary care and any procedural services (eg, colonoscopy or sigmoidoscopy) at the institution for insurance reasons, so we were confident, based on prior analyses of this database, that nearly all episodes of screening would be captured.

### Variables

The dependent variable, colorectal cancer screening, was defined as having a documented episode of either colonoscopy or sigmoidoscopy between January 1, 2005, and January 1, 2010, or FOBT between January 1, 2009, and January 1, 2010 (the data were assembled in May 2010). We also conducted sensitivity analyses with 10-year screening rates of colonoscopy for patients who were aged 50 years or older on January 1, 2000. We chose the 5-year rather than the 10-year screening variable for our primary analyses because we felt that other variables of interest, notably HbA1c and number of primary care visits, would be less consistent over a longer time period. The independent variable of interest, glycemic control, was defined as the average HbA1c between January 1, 2005, and January 1, 2010. For the bivariate analyses, we defined good control as HbA1c less than or equal to 7.0%, fair as HbA1c 7.0% to 8.5%, and poor as HbA1c greater than 8.5%. For the logistic regression, these categories were collapsed into good (≤8.5%) and poor (>8.5%).

Several covariates were also analyzed: age (continuous), sex, race/ethnicity (patients self-identify at registration and we collapsed the many subcategories into white, black, Latino, or other to be consistent with other studies), language preference (English or non-English, indicated by the patient during registration), and number of primary care visits in 5 years (assuming the ideal is 4 visits per year for diabetic patients, we categorized the number of visits as low [<20], ideal [20-30], and high [>30]). We also examined insurance status at the beginning of the 5-year period (government insurance, private insurance, or no insurance); emergency department (ED) visits in the 5-year period (<10, 10-20, and >20); no-shows for clinical appointments in the 5-year period (continuous); and incidence of end-stage renal disease (yes or no), which can complicate preparations for colorectal cancer screening.

### Statistical analysis

We performed bivariate analyses using χ^2^ tests to determine which variables were associated with lack of screening and with good, fair, and poor HbA1c averages. Correlation coefficients were used to confirm the relationship between primary care visits, ED visits, and no-shows. Finally, logistic regression was employed to determine the independent association of variables of interest with colorectal cancer screening. We selected our model by sequentially removing variables from the model and leaving them out if their removal did not change any odds ratios by 10% or more. We wanted to show any potentially interesting associations, but our large database could result in some associations being statistically significant but clinically insignificant. Consequently, the final model contains only variables that 1) were significant in the initial model with respect to colorectal cancer screening and 2) when removed from the model, changed other associations by 10% or more. SAS version 9.1 (SAS Institute, Inc, Cary, North Carolina) was used for all analyses.

## Results

The study population was 58% female, 57% black, and 14% Latino (Table 1). The colorectal cancer screening rate overall was 38%. Most patients (59%) were covered by some form of government insurance; 22% had no listed insurance and 19% had private insurance. Thirty-six percent of patients had good glycemic control, 37% fair control, and 27% poor control.

Average HbA1c was strongly associated with screening; the highest rates of screening occurred in patients whose HbA1c was 8.5% or less (Table 1). We observed significant associations for race/ethnicity; patients with end-stage renal disease also had higher screening rates than those without. Higher numbers of primary care visits were strongly associated with screening, as expected. However, more frequent ED visits also appeared to be strongly associated with screening.

On average, patients with poor (>8.5%) HbA1c values tended to be younger, uninsured, nonwhite, and have higher rates of no-shows compared with patients exhibiting good and fair HbA1c values (Table 2). The number of primary care visits did not appear to be lower in the group with high HbA1c values.

Correlation coefficients indicated that primary care visits were weakly correlated with ED visits (ρ = 0.19) and no-shows (ρ = 0.34). Although the Pearson and Spearman coefficients were similar, we report the Spearman coefficients because the numbers of primary care visits, ED visits, and no-shows were not normally distributed.

We also performed sensitivity analyses to examine different types of colorectal cancer screening individually by HbA1c level. We found that for all screening groups (5-year colonoscopy, 5-year sigmoidoscopy, 1-year FOBT, and 10-year colonoscopy) the rates of colorectal cancer screening decreased as the glycemic control worsened.

In the final logistic regression model, poor average HbA1c was predictive of not receiving colorectal cancer screening, as was having had fewer than 20 primary care visits in a 5-year period (Table 3). Patients with either government or private insurance were more likely to have been screened than those with no insurance.

## Discussion

Poor glycemic control was associated with not being screened for colorectal cancer, even after adjusting for number of primary care visits and for insurance status. This finding suggests that the issue for these patients goes beyond simply not being seen in primary care offices; there may also be issues of competing priorities (19,20) in the office visit when they do go. Either physicians occupied with helping their patients to gain better glycemic control may be less likely to recommend colorectal cancer screening, or patients struggling to gain better glycemic control may be less likely to follow through on the recommendation. Other studies on cancer screening in people with diabetes (16-18) have also noted lower rates of screening and proposed similar hypotheses (both patient and physician factors) for them. Further research, with primary data collection, would be ideal to define barriers to colorectal cancer screening specifically for people with diabetes.

The emerging research on colorectal cancer risk for people with diabetes suggests that diabetic patients with poor glycemic control may have even higher risk because they exhibit high levels of circulating insulin and IGF-1 and are often receiving multiple treatments (21-25). Therefore, people with poorly controlled diabetes are an important group to target for colorectal cancer screening, even if the care providers are spending most of their time on diabetes management.

This study had several limitations. Although the database's information was extracted from electronic medical records and was complete for most variables, we could not obtain reliable data on several variables of interest: years since diabetes diagnosis, type of diabetes, other diabetic complications beyond end-stage renal disease, reason for colonoscopy (screening vs diagnostic), and physician recommendation for colorectal cancer screening. These are all variables that should be examined further for associations with colorectal cancer screening among people with diabetes. The data also came from a large, urban, safety-net hospital with large numbers of black and Latino patients. We considered this a strength in terms of obtaining a robust sample of racial/ethnic minorities for analyses, but the association of both groups with higher colorectal cancer screening rates (compared with white patients) may not be generalizable to other settings.

We hope the findings of this study will help to provide context for the recent studies associating diabetes with a higher risk of colorectal cancer and polyps; this information is more valuable if more is known about the colorectal cancer screening patterns of people with diabetes and the specific factors associated with lack of colorectal cancer screening for this group. Further studies are needed to explore this finding and to determine the association of other variables with colorectal cancer screening for people with diabetes. Future research could specifically examine variables related to the hypotheses we generated about low colorectal cancer screening rates in people with poor glycemic control but adequate numbers of primary care visits, to define whether patient factors or physician factors are more closely related to the lack of colorectal cancer screening. This information might help health care systems to promote screening in this group of patients, which appears to be more at risk for colorectal cancer and more at risk for not being screened.

## Figures and Tables

**Table 1:** Associations of Health Care Use and Demographic and Health Characteristics With Receiving Colorectal Cancer Screening^a^, Boston, Massachusetts, 2005-2010^b^

Characteristic	n	% Who Had Colorectal Cancer Screening	% With No Colorectal Cancer Screening	*P* Value^c^
**Age, mean (SD), y**	6,066	62.5 (8.6)	64.3 (9.6)	<.001^d^
**Sex**				
Male	2,562	38.7	61.3	.43
Female	3,504	37.7	62.3	
**Race/ethnicity**				
White	984	37.5	62.5	<.001
Black	3,456	39.0	61.0	
Latino	835	38.8	61.2	
Other	791	34.8	65.2	
**Language preference**				
English	4,198	39.7	60.3	.43
Other	1,868	34.6	65.4	
**End-stage renal disease**				
Yes	430	46.5	53.5	<.001
No	5,636	37.5	62.5	
**No. of ED visits in 5 years**				
<10	3,845	38.6	61.4	<.001
10-20	568	50.7	49.3	
>20	193	59.1	40.9	
None/no data available	1,460	29.4	70.6	
**Mean no. of no-shows for 5-year period (SD)**	9,109	9.0 (9.2)	7.1 (7.6)	<.001^d^
**Health insurance coverage**				
Government	3,579	40.4	59.6	<.001
Private	1,156	43.0	57.0	
None	1,331	28.0	72.0	
**No. of primary care visits in 5 years**				
>30	532	61.1	38.9	<.001
20-30	1,287	49.4	50.6	
1-19	3,722	32.4	67.6	
0	525	27.8	72.2	
**Mean no. of HbA1c tests (SD)**	9,059	8.4 (4.4)	5.9 (4.5)	<.001^d^
**Mean HbA1c, %^e^**				
<7.0	2,184	40.3	59.7	<.001
7.0-8.5	2,267	41.1	58.9	
>8.5	1,605	31.2	68.8	

Abbreviations: SD, standard deviation; ED, emergency department; HbA1c, hemoglobin A1c.

a Defined as colonoscopy or sigmoidoscopy within 5 years or fecal occult blood test within 1 year.

b All patients in sample were aged ≥50 y and had been diagnosed with diabetes. Values are percentages unless otherwise indicated.

c Calculated by using χ^2^ tests, except where indicated by footnote d.

d Calculated by using independent *t* tests.

e Total for this variable is not 6,066 because of missing values.

**Table 2:** Associations of Health Care Use and Demographic and Health Characteristics With Glycemic Control, Boston, Massachusetts, 2005-2010^a^

Characteristic	n	Mean HbA1c <7.0%	Mean HbA1c 7.0%-8.5%	Mean HbA1c >8.5%	*P* Value^b^
**Age, mean (SD), y**	6,066	65.2 (10.0)	63.7 (9.1)	61.5 (8.3)	<.001^c^
**Sex**					
Male	2,562	35.5	37.0	27.3	.57
Female	3,504	36.4	37.7	25.8	
**Race/ethnicity**					
White	984	47.1	36.4	16.6	<.001
Black	3,456	34.1	37.4	28.2	
Latino	835	33.3	36.8	29.8	
Other	791	33.4	39.2	27.4	
**Language preference**					
English	4,198	38.7	36.6	24.6	<.001
Other	1,868	29.9	39.2	30.7	
**End-stage renal disease**					
Yes	430	39.5	37.7	22.8	.19
No	5,636	35.7	37.4	26.7	
**No. of ED visits in 5 years**					
<10	3,845	34.8	37.6	27.4	<.001
10-20	568	37.7	34.0	28.4	
>20	193	37.8	37.8	24.4	
None/no data available	1,460	38.2	38.2	23.6	
**Mean no. of no-shows for 5-year period (SD)**	9,109	6.5 (7.4)	7.2 (7.4)	9.3 (9.7)	<.001^c^
**Health insurance coverage**					
Government	3,579	37.2	38.1	24.6	<.001
Private	1,156	38.3	35.9	25.6	
None	1,331	30.8	36.7	32.2	
**No. of primary care visits in 5 years**					
>30	532	31.4	41.2	27.4	<.001
20-30	1,287	35.0	43.3	21.5	
1-19	3,722	35.6	35.4	28.8	
0	525	45.9	32.8	21.3	
**Mean no. of HbA1c tests (SD)**	9,059	6.1 (4.3)	7.6 (4.6)	6.9 (4.9)	<.001^c^

Abbreviations: HbA1c, hemoglobin A1c; SD, standard deviation; ED, emergency department.

a All patients in sample were aged ≥50 y and had been diagnosed with diabetes. Values are percentages unless otherwise indicated.

b Calculated by using χ^2^ tests, except where indicated by footnote c.

c Calculated by using the *F* statistic with generalized linear models.

**Table 3:** Odds of Not Receiving Colorectal Cancer Screening^a^, by Health Care Use and by Demographic and Health Characteristics, Boston, Massachusetts, 2005-2010^b^

**Characteristic**	OR (95% CI)	Adjusted OR (95% CI)
**Glycemic control**		
HbA1c >8.5%	1.51 (1.34-1.71)	1.56 (1.38-1.77)
HbA1c ≤8.5%	1 [Reference]	
**Sex**		
Male	1 [Reference]	
Female	1.04 (0.94-1.16)	NC
**Race/ethnicity**		
White	1 [Reference]	
Black	0.92 (0.83-1.03)	NC
Latino	0.97 (0.83-1.13)	NC
**No. of primary care visits in 5 years**		
≥20	1 [Reference]	
<20	2.40 (2.14-2.68)	2.19 (1.95-2.46)
**No. of ED visits in 5 years**		
0-10	1 [Reference]	
>10	1.05 (0.94-1.17)	NC
**Age, y^c^**	1.02 (1.02-1.03)	1.03 (1.02-1.03)
**End-stage renal disease**		
Yes	0.69 (0.57-0.84)	NC
No	1 [Reference]	
**Language preference**		
English	1 [Reference]	
Other	1.25 (1.11-1.40)	NC
**Health insurance coverage**		
Government	0.79 (0.71-0.88)	0.70 (0.60-0.80)
Private	0.78 (0.68-0.89)	0.68 (0.57-0.81)
None	1 [Reference]	

Abbreviations: OR, odds ratio; CI, confidence interval; HbA1c, hemoglobin A1c; NC, not calculated; ED, emergency department.

a Defined as colonoscopy or sigmoidoscopy within 5 years or fecal occult blood test within 1 year.

b All patients in sample were aged ≥50 y and had been diagnosed with diabetes.

c Any selected age (≥50 y) can be used as the reference group. For example, to estimate the odds of not receiving colorectal cancer screening among people aged 70 y with people aged 50 y, subtract 50 from 70 and raise the OR (1.02) or adjusted OR (1.03) to that power.
